# The impact of neighborhood environment and social interaction on the health of Chinese residents: empirical analysis from CGSS 2021

**DOI:** 10.3389/fpubh.2025.1547499

**Published:** 2025-05-07

**Authors:** Dongliang Wang, Huanjun Li, Ping Liu

**Affiliations:** ^1^School of Law, Chongqing University, Chongqing, China; ^2^School of Ethnology and Sociology, Minzu University of China, Beijing, China; ^3^School of Pediatrics, Chongqing Medical University, Chongqing, China

**Keywords:** neighborhood environment, social environment, physical environment, social interaction, the health of residents

## Abstract

**Background:**

The neighborhood environment plays a crucial role in shaping residents’ health and quality of life.

**Methods:**

Based on 2021 Chinese General Social Survey (CGSS 2021), we used multiple linear regression model to conduct regression analysis and mediation analysis, focusing on the relationship among neighborhood environment, social interaction and residents’ health. Our sample size is 8148 people, including 3679 men and 4469 women, 5412 in urban areas and 2736 in rural areas, the mean age was 54.64 years.

**Results:**

We find that the mean score of Chinese residents’ health is 2.88, which is above the medium level, and the neighborhood environment, social environment and physical environment have significant positive effects on the health of residents. Social interaction plays a mediating role in the mechanism of social environment on residents’ health, whereas it plays a moderating role in the relationship between the physical environment and health. Distinctions in health outcomes are observed between urban and rural residents as well as between individuals institutional/non-institution employment, highlighting the influence of social environment disparities.

**Conclusion:**

Our research underscores the importance of improving neighborhood environment, fostering social interaction and implementing inclusive public policies to promote equitable health outcomes.

**Discussion:**

Future research is recommended to explore more refined mechanisms linking neighborhood environment and health through longitudinal designs and experimental interventions.

## 1 Introduction

Health is the foundation of life activities and an important goal of comprehensive human development, not only important for personal well-being, but also a key indicator of economic development and social governance (1). However, when people enter the modern society, the high speed and fast pace of lifestyle and workstyle pose a threat to people’s health (2). Accordingly, how to improve the health status of residents and improve the quality of life of the older adult continue to attract attention (3). Especially since the 2020s, many countries have gradually entered the aging society or the aging problem has been deepening (4) while the health problem of residents has become a major social problem that needs to be faced and solved urgently (5). The COVID-19 outbreak at the end of 2019 affected the world and spread to the present, which has made the impact of neighborhood environment on residents’ health become the focus of attention in various countries and regions (6). The World Environmental Convention under negotiation is intended to make environmental right a third-generation fundamental human right to promote justiciability and environmental accountability, thereby improving the health of the population (7).

Health has been a major concern of scientists since a long time ago. With the increasing level of economic development and the diversification of lifestyles, the health of residents is no longer studied only as the content of medicine, in which health is regarded as the result of the influence of viruses, bacteria, and genes, but is increasingly influenced by the external social and spatial environment (8). Health is increasingly becoming a social issue. Community environment is the basic unit of residents’ life and there is a correlation between it and residents’ health (9). It is also an important means to improve the health literacy of residents (10). Community environment is the sum of the natural, humanistic and economic conditions on which the residents depend for their survival and the generation of neighborhood activities (11), including built environment, natural environment and social environment (12). The built environment is the variety of buildings and places constructed and modified by human beings (13), consisting of a range of elements such as land use, transport organization and spatial design (14), which are closely related to the physical activities (15, 16), social capital (17), modes of transport and pro-natural behavior of residents (18) and its correlation with the physical and mental health of residents has been identified (19). The natural environment is related to natural ecology, and the current focus is on the areas such as neighborhood green spaces (20, 21), and neighborhood parks (22). The social environment focuses on the soft strengths of the neighborhood, such as neighborhood safety, neighborhood health care and neighborhood relationships, which have an obvious role in promoting the mental and social health of residents (23). The natural environment has the greatest direct impact on residents’ health and the physical environment has the greatest indirect and comprehensive impact on residents’ health (24). Some scholars also study the community environment as a moderating effect (25, 26).

In this context, the impact of the neighborhood environment on the health of residents is an emerging area of research in the 21st century (27). Researches have focused more on the impact of neighborhood environment on physical activities in adolescents (28), recreational walking (29) and physical activity (30), dietary intake (31) and the health of the older adult (32). The results of studies linking the demographic characteristics of neighborhoods to the health of their residents are mixed, due to issues such as the specific scope of the neighborhood and the censuses, which complicates comparisons between studies, but there are some consistent conclusions that can be drawn from them. In general, most studies show that there is a strong correlation between the quality of the neighborhood environment and the health of residents, with good neighborhood environments improving the health of residents, and poor neighborhood environments discouraging the healthy behaviors and leading to lower levels of health. However, the influence of the neighborhood social environment on the health of residents is more in the area of mental health, and less research has been conducted on the impact on physical health. Therefore, the innovation of this study is to systematically analyze the influence mechanism of social environment and physical environment on residents’ health in the neighborhood environment based on the data of 2021 Chinese General Social Survey (CGSS 2021), especially the mediating effect and moderating effect of introducing social interaction variables. In addition, this paper also distinguishes urban–rural differences and deepens the understanding of the impact of neighborhood environment on the health of different groups, which is the first time in the existing literature.

## 2 Literature review and theoretical hypothesis

### 2.1 The relationship between community environment and residents’ health

Community environment is an important predictor of residents’ health status (33). As an important site for interpersonal communication, the neighborhood provides residents with opportunities for social interaction, enhances neighborhood cohesion among residents, and plays a mediating role between neighborhood interaction and physical and mental health (34). Through a study of community health educators, neighborhood was a contextual factor that influenced the outcome of health interventions and related concepts were social networks, social embeddedness, social capital (35). Bevan et al. focused on the relationship between the social environment and the health of residents, and concluded that social cohesion, social networks, social support and social capital influenced the health of residents (36). Some studies have developed that social capital and neighborhood cohesion are associated with residents’ self-reported health (37), affecting residents’ BMI and obesity (38, 39). Soc-environmental factors such as neighborhood safety and social disorder have also been linked to the health status of residents (40, 41). The neighborhood barriers affect residents’ health behaviors and health outcomes in three ways: by encouraging risky behaviors, discouraging physical activities and creating psycho-social stressors (42). More importantly, residents’ perceptions of neighborhood environment are foundational in influencing health and moderating the neighborhood environment (43).

Roux and Mair further categorize neighborhood environment into two types: the neighborhood physical environment and the neighborhood social environment (44). The neighborhood physical environment affects the travel behaviors and sports activities of residents (45). Commercial accessibility is closely related to residents’ eating habits and food quality (46). Factors such as the geographical location, socio-economic status and level of economic development of a neighborhood are usually closely related to the health status of its residents. Those living in the poorer environments and economically deprived neighborhoods have a lower level of self-reported health, a higher likelihood of mental illness, and a higher risk of developing a variety of diseases (47, 48). Among them, the physical environment of the community mainly affects the mental health of the floating population in the old community, while the social environment plays a decisive role in the mental health of the floating population in the new community (49). Poor community social environment, including lack of social support, social network and social cohesion (50), may lead to lack of physical activity (51); while the improvement of the community environment may curb the growth of obesity (52).

Some scholars also believe that the community social environment has a stronger impact on the mental health of residents than the built environment (53). Community parks, as important neighborhood activity spaces, have a significant impact on the health of residents, which increase their interest in physical activities, extend the time spent outdoors, and thus improve their health (54). Moreover, it has been confirmed by Chinese scholars that community greening can improve residents’ self-rated health by alleviating psychological pressure (55). Hughey et al. illustrated the same finding from a social justice perspective (56). As inclusive places, neighborhoods are increasingly being built with an emphasis on the quality of the environment and the level of amenities. Improving the amount and quality of green spaces in the neighborhood could increase the physical activities of residents, which had a benign effect on the physical health (57). Meanwhile, a good physical environment in the neighborhood provides a suitable place for residents to engage in social activities, which helps to improve the cohesion of the neighborhoods and the social support systems, and alleviates residents’ loneliness and depression, and promotes the mental health (58). Housing, an important foundation of the neighborhood, also contains a range of physical, social, and psychological factors, all of which have an impact on the health of residents, and housing stability has therefore been identified as an important indicator of neighborhood health (59). Community environment may also be unrelated to residents’ health (60). In addition, the impact of community environment on the health of residents may also be different. For example, the urban community environment has a significant positive effect on the mental health of the older adult, while the rural community environment has a negative impact on the mental health of the rural older adult (61). Compared with the local residents, the community environment and the health of the floating population are more closely related (62).

### 2.2 The influence of neighborhood environment on residents’ health

The neighborhood environment is an important factor affecting residents’ health and physical function (63). Some studies have found that there are significant differences between the ways in which the neighborhood environment affects mental health: the social environment plays a mediating and moderating role between the built environment and mental health, but the built environment has a more significant impact on mental health (64). The neighborhood environment perception has an independent and significant impact on residents’ self-rated health, while the influence of neighborhood social environment perception on residents’ self-rated health was more significant (65).

There are two main views about the effect of neighborhood environment on residents’ health. The first is that the neighborhood environment can effectively improve the residents’ health (66). The neighborhood environment improves residents’ living conditions, travel convenience and service accessibility, which in turn improves the overall residents’ health (67). This view is the mainstream view in academia. Because the health status of residents living in a good community environment is better (68) and there is a strong relationship between the physical and social environment of the neighborhood and the overall health status of rural residents (69). The unfavorable neighborhood environment may cause malignant stimulation to residents’ health and indirectly affect residents’ mental health (70). The physical environment encompasses both natural and built characteristics. The natural environmental factors (residents’ perceptions of air quality and water pollution in their living environment) will also have impacts on residents’ health status (71). For example, the neighborhood physical environment such as noise and environmental pollution will reduce the residents’ physical and mental health (72–74). Conversely, sound public facilities and perfect medical services can make residents have a good level of exercise and easier access to medical assistance, which can further reduce the incidence of chronic diseases and improving their physical and mental health (75, 76). The second view emphasizes the ineffectiveness of community environment on improving residents’ health, which reflects that the impact of neighborhood environment on residents’ physical health may be overestimated and the secondary role of neighborhood environment in explaining people’s mental health (53). Through a three-year follow-up of the older adult over 60 years old, it was found that the influence of neighborhood environmental factors on the changes of skeletal muscle mass index (SMI) and grip strength of the rural older adult was limited (77). The trust relationship between neighbors was associated with mental health but not with physical health (78). In addition, the effect of neighborhood-built environment index on residents’ physical and mental health status may be weak (79).

The impact of the neighborhood environment on residents’ health may vary by group, age, gender and even country. The association between social environment and self-rated health is stronger in Japan than in China and South Korea (80). Individuals who lived in disadvantaged community environment and exhibited unhealthy behaviors had the greatest increases in mortality (81), while residents who were dissatisfied with their overall community environment were more likely to have negative views on health and depressive symptoms (82). Older people living in disadvantaged communities have more physical health problems than those living in strong communities (83). Compared with adolescence, neighborhood environment seems to have a long-term effect on BMI (Body Mass Index) in adulthood (84). High-quality neighborhood environment can promote the physical and mental health of migrant adolescents (85). Adolescents’ physical health is influenced by the characteristics of the community-built environment and school neighborhood, while the factors of school neighborhood environment are more important to adolescents’ physical health (86). Outdoor activities have the greatest impact on middle-aged groups (70–79 years) and younger age groups (60–69 years) (87). In terms of the impact of neighborhood environment on childhood obesity, boys are less sensitive to neighborhood environment than girls (88).

It can be seen that most of the existing studies have investigated the impact on health from a single neighborhood environment, and the target group of the studies is mostly focused on a specific age group, and we suggest that the study on the impact of the integrated neighborhood environment on the health of residents of all ages needs to be deepened. Combining the results of existing studies, we consider the social and physical environment of the neighborhood, take Chinese urban and rural residents as research subjects, and put forward the hypothesis that the social and physical environment of neighborhood could affect the health of residents. Hypothesis 1 and Hypothesis 2 are presented as follows:

Hypotheses 1. The better the neighborhood social environment, the higher the level of the health of residents.

Hypotheses 2. The better the neighborhood physical environment, the higher the level of the health of residents.

### 2.3 The influence of social interaction on residents’ health

Social interaction is an important indicator that affects residents’ health (89) and it is considered to be the main practice type to meet residents’ spiritual needs (90). Both individual informal social interaction and formal group participation have a significant impact on residents’ health (91). However, group interaction can reduce the incidence of depression, while individual interaction tends to have complicated effects on the mental health of migrant workers (92). Cable et al. used friend socializing and relative socializing to measure the impact of social interaction on the mental health of civil servants (93). Social interaction with friends is one of the most important social activities to improve the mental health of middle-aged and older adult people (94). The frequency of social interaction (95) and meeting with friends is significantly positively correlated with the health status of residents (96). People who experience a lot of social interaction tend to be less stressed and the number of social interactions is related to personal mental health (97). On the one hand, social interaction can form a beneficial social environment in the resident group, reduce individual stress and thus reduce the occurrence of unhealthy behaviors (98, 99). Social activity participation plays a moderating effect in the influence of community environment on the occurrence of possible sarcopenia in people aged 45 years above (100). From young age to old age, the influence path of social interaction on the health of the older adult presents the characteristics of transferring from individual behavior to community interpersonal communication (101); while the older adult with close social networks have better self-rated health status than those who are more isolated in daily life (102). It should be noted that although social interaction with neighbors affects the health of the older adult (103). However, family companionship had significantly greater positive effects on the health of older adults than the companionship of friends (104). On the other hand, social isolation and non-supportive social interaction can lead to decreased immune function and increased neuroendocrine and cardiovascular activities (105). In particular, the adverse effects of personal financial difficulties on changes in self-rated health status are more pronounced when the level of negative social interaction gradually increases (106).

Community support has significant positive impacts on residents’ health (107), while social support plays a mediating role between social participation and self-rated health (108), neighborhood relationship and subjective well-being (109). The number of community organizations and the frequency of neighborhood interaction have significant positive predictive effects on mental health, while the number of community disputes has significant negative predictive effects on mental health (110). Residents living in well-connected neighborhoods have better physical and mental health because they are more likely to engage in social interaction and avoid loneliness (111, 112). For example, the community social interaction environment has significant positive effects on the mental health of the urban older adult, but it has negative impacts on the mental health of the rural older adult (61). Face-to-face communication can promote the individual’s mental health level more than non-face-to-face communication (113). However, the impact of social interaction on residents’ physical and mental health has obvious intergenerational and gender differences. On the one hand, social interaction has significant positive impacts on the physical health of the older generation of migrant workers and has more significant impacts on the mental health of the new generation of migrant workers (114). On the other hand, social interaction has significant positive impacts on the health self-rated of older adult women and men get more health benefits from relatives and friends than women (95). The collective social interaction of participating in folk-belief activities has no significant impact on male health, but only has a significant impact on the health of rural women when the frequency of participation is high and the impact is lower than that of rural women (115). In addition, voting in community council elections can affect the health of residents, but the direction of this effect has not been clearly confirmed (116). For example, participation in grass-roots elections and community engagement had no influence on the population’s health. Nevertheless, political engagement that took place outside the system, like struggling for rights, had significantly negative effects on the mental health of the population (117). Participation in volunteer service activities can significantly affect the health status of residents (118). With the increase of voluntary positive behavior, the individual health level of residents will be improved more significantly (119). However, there is not enough evidence to prove that the type or intensity of voluntary service has a consistent impact on residents’ health (120) and how much impact volunteer service has on residents’ health remains to be determined (121).

In summary, neighborhood social interaction has a significant impact on residents’ physical and mental health, while the direction of the impact has not been unanimously confirmed. However, the mutual affirmation during social interaction promotes mental health and reduces the tendency to depression (122). Mini-mental state examination, self-retained depression scale and general self-efficacy scale are also significantly associated with informal social interaction and formal group participation (91). Conversely, inadequate social interaction and social relationships have a negative impact on mental health (123). While the good neighborhood environment can promote social interaction and interpersonal relationships among neighbors and increase the sense of neighborhood identity (101). In the context of Chinese society, urbanization has led to the loosening of social ties and the less interaction and communication among urban residents, and that a slow-paced, low-density neighborhood environment is needed to foster neighborhood trust and social interaction (124). This leads to the following hypotheses:

Hypothesis 3. The better the neighborhood social environment is positively correlated with the higher the level of the residents’ health, mediated through the residents’ social interaction.

Hypothesis 4. The better the neighborhood physical environment is positively correlated with the higher the level of the residents’ health, mediated through the residents’ social interaction.

**Figure 1:**
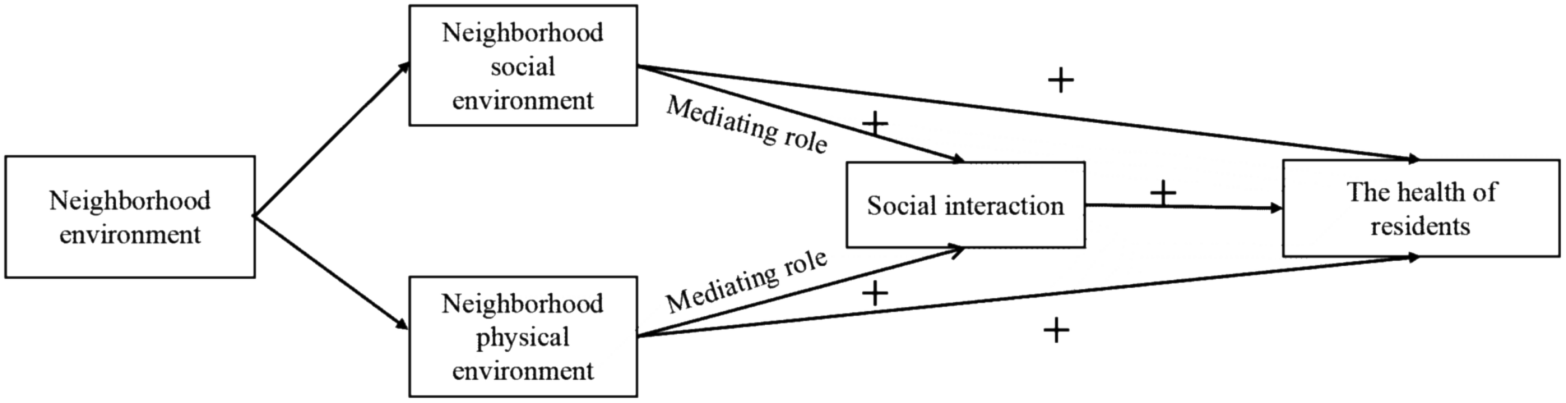
The relationship between neighborhood environment, social interaction and the health of residents.

In theory, the neighborhood environment has a direct or indirect effect on health by affecting residents’ daily behavior and social relations. Figure 1 shows the theoretical model of this study, including: (1) Neighborhood social environment directly affects health; (2) Neighborhood physical environment directly affects health and; (3) Social interaction plays an intermediary role in the above relationship. The predicted graph of the relationship between neighbourhood environment, social interaction and the health of residents is as follows:

## 3 Data and research strategy

### 3.1 Data and methods

This study uses the data from the 2021 Chinese General Social Survey (CGSS), a survey conducted by the China Survey and Data Centre of Renmin University of China, which covers more than 10,000 households in all provinces, municipalities directly under the central government and autonomous regions of mainland China. And the CGSS focuses on a wide range of issues at multiple levels of society, community, family and individual. In the CGSS2021 survey, a total of 8,148 people responded to the questionnaire, of which 3,679 were male, accounting for 45.15%, and 4,469 were female, accounting for 54.85%. The number of married people was 6,007, or 73.72%, and the number of unmarried people was 2,141, or 26.28%. The urban population was 5,412, or 66.42%, and the rural population was 2,736, or 33.58%. The age of population ranged from 21 to 102 years, with an average of 54.64 years.

The purpose of this study is to investigate whether and how different neighborhood environments affect the health of residents and the mediating mechanisms of social interaction. First, this study used multiple linear regression models and Stata MP 17 statistical software to analyze the data and investigate the relationship between neighborhood environment and the health of residents. Second, the variable of social interaction was added to analyze the intermediate role in the influence chain of “neighborhood environment-the residents’ health.” In order to control the possible bias caused by the uneven proportion of urban and rural residents, this study uses a weighted method to adjust the data to make the urban and rural population ratio more consistent with the actual distribution of 2021 China Statistical Yearbook. In addition, the variables with many missing values in the data were interpolated to reduce sample bias. In this way, it would be possible to verify the mechanisms of influence that exist among the neighborhood environment, social interaction and the health of residents.

### 3.2 Dependent variable

The dependent variable in this study is the health of residents. And the self-reported health of residents was chosen as the indicator to measure the health of residents in this study, because the health was very complex and had a wide range of scopes and definitions, and residents’ knowledge of their own health status was more comprehensive and integrated. The question in the questionnaire was worded as: “How do you think your health is at the moment?” The answers were categorized into five levels according to the Likert scale: 1 = very good, 2 = good, 3 = average, 4 = poor, 5 = very poor. In order to facilitate the subsequent statistical analysis, this study adjusted the way in which the dependent variable was assigned, with the health status of residents taking the value from 1 to 5 from low to high, and the missing values were eliminated.

### 3.3 Independent variable

In this study, the neighborhood environment variables are derived from the following items of the questionnaire: “Is the place of residence suitable for exercise,” “Is there a sufficient supply of fresh fruits and vegetables,” “Is public facilities sufficient,” “Neighborhood safety,” “Neighborhood mutual care” and “Neighborhood mutual willingness.” The result of KMO test was 0.655, Bartlett test was significant (*p* < 0.001), which indicates that it was more suitable for factor analysis (Table 1). On this basis, two principal components were extracted, named social environment and physical environment, respectively, and the cumulative variance interpretation rate was 62.2% (Table 2).

**Table 1:** KMO indicator results.

KMO	0.655	
Bartlett test of Sphericity	Chi-square	3849.630
	Degrees of freedom	15
	*p*-value	0.000

**Table 2:** Items and component matrices in the neighborhood environment.

Items	Means	SD	Rotated factor component matrix	
			The social environment factor	The physical environment factor
Be suitable for physical exercise	3.83	1.01		0.7372
Be available for vegetables and fruits	4.10	0.83		0.6678
Enough public facilities	3.18	1.25		0.7429
Safe	4.19	0.71	0.4895	
Take care of each other	3.94	0.87	0.9115	
Be willing to help	4.01	0.80	0.9109	
Variance			1.9814	1.7509
Cumulative			62.20%	
Method			Principal—component factors	

### 3.4 Moderating variable

The moderating variable in this study is social interaction. The question selected for treatment was: “How often do you engage in social and recreational activities with your neighbors?” and the answers were categorized into seven degrees: 1 = almost every day, 2 = once or twice a week, 3 = a few times a month, 4 = once a month, 5 = a few times a year, 6 = once a year or less, and 7 = never. The missing values were eliminated and the values were assigned in reverse order ranging from 1 to 7.

### 3.5 Control variables

We controlled for demographic and contextual variables this study, mainly gender (male = 1; female = 0), age, marital status (married = 1; unmarried = 0), education level (high education = 1; other = 0), political profile (party member = 1; other = 0), character of occupation (institutional = 1; non-institutional = 0), and place of residence (urban = 1; rural = 0). Given the particular social context of China, the classification of occupation largely implied institutional segmentation (or institutional/non-institutional employment) (125), so the study took this variable into account as well, classifying the character of occupation as institutional or non-institutional. Considering the high level of social mobility in China, most residents with rural Hukou came to the city for work, and their place of residence and usual social interaction took place in the city, meaning that the Hukou cannot accurately differentiate between the living conditions of residents, so the place of residence was chosen as a control variable instead of Hukou.

## 4 Results

### 4.1 Descriptive statistical analysis

Table 3 shows the basic situation of the residents’ health in Chinese society. Overall, the average score of Chinese residents’ health is 2.88, which is located at the middle-upper level, meaning an urgent need for further improvement in the health of residents in China. There is a positive correlation between the neighborhood environment and the health of residents, with the correlation coefficient between the physical environment of neighborhood and the health of residents being greater than that between the social environment and the health of residents. The health level of males is higher compared to females and higher than the overall mean. The unmarried population is much healthier than the average, while the married population is less healthy. Age is inversely related to the level of health, with a gradual decrease in the health with increasing age. Educational attainment is positively correlated with the health. Those with higher and secondary education have a significantly better health than those with only lower education, whose health level is well below the average. It is consistent with the findings of existing studies. As an important indicator of socio-economic status, educational attainment clearly shows a positive predictor for high socio-economic status to high levels of the residents’ health. Party members are healthier than non-party members. The health of those with institutional occupations is also much better than that of those working outside the institution. According to the results of some existing studies, the possible explanations are: on the one hand, institutional occupations generally mean stable income and rich welfare, which are economically advantageous in improving the physical health (126); on the other hand, institutional occupations provide residents with a higher social status and a richer set of social network resources, which play a useful role in maintaining the mental health (127). In the meantime, the urban population is healthier than the rural population, being much less healthy than the average.

**Table 3:** Descriptive statistical analysis of the health of residents.

Variables		The health of residents
		Mean (SD)/corr.
The health of residents		2.88 (1.23)
Neighborhood environment	The physical environment	0.1176***
	The social environment	0.0104***
Gender	Male	2.94 (1.26)
	Female	2.83 (1.21)
Marital status	Married	2.84 (1.21)
	Unmarried	3.02 (1.29)
Age		−0.3479***
Education	Higher education	3.42 (1.10)
	Secondary education	2.91 (1.22)
	Lower education	2.53 (1.21)
Political profile	Party member	3.01 (1.23)
	Non-party member	2.86 (1.23)
Occupation	Institutional	3.20 (1.11)
	Non-institutional	2.86 (1.24)
Place of residence	Urban	2.98 (1.21)
	Rural	2.70 (1.26)

*p < 0.05, **p < 0.01, ***p < 0.001.

### 4.2 Regression analysis

The study used a multiple linear regression model to identify the relationship between neighborhood environment and the health of residents (Table 4). Model 1 introduced control variables, and the results showed that gender, age, education, political profile, and place of residence all had a significant impact on the health of residents. Specifically, males are healthier than females, those with higher education are much healthier than the rest of the educated population, party members are healthier than non-party members, and urban residents are healthier than rural residents, and all of the significance is high (*p* < 0.001). With the increase of age, the health level of residents decreases significantly. We added the first independent variable the social environment in Model 2, and we found that there was a significant positive correlation between the social environment and the health of residents (*p* < 0.001), and every 1-point increase in the social environment increases the health of residents by 0.126 points. Model 3 added the second independent variable the physical environment on the basis of Model 1, and the results were still significant. For every 1-point increase in the physical environment, the health of residents increases by 0.104 points. Hypotheses 1 and Hypothesis 2 were verified. The coefficients of gender, age, education, political profile and place of residence are equally significant in Model 2 and Model 3.

**Table 4:** Results of multiple regression analysis (impact of neighborhood environment on residents’ health).

Variables	Model 1	Model 2	Model 3
Gender (male)	0.117** (0.045)	0.123** (0.045)	0.116** (0.045)
Marital status (married)	0.001 (0.052)	−0.003 (0.052)	−0.009 (0.053)
Age	−0.022*** (0.002)	−0.023*** (0.002)	−0.023*** (0.002)
Education (higher)	0.199** (0.082)	0.233** (0.084)	0.167* (0.085)
Political profile (party member)	0.169* (0.074)	0.165* (0.073)	0.162* (0.073)
Occupation (institutional)	−0.068 (0.093)	−0.082 (0.092)	−0.072 (0.092)
Place of residence (urban)	0.145** (0.050)	0.194*** (0.050)	0.100* (0.050)
The social environment		0.126*** (0.023)	
The physical environment			0.104*** (0.023)
F	52.69	50.72	49.48
p	0.000	0.000	0.000
R^2^	0.135	0.144	0.141

*p < 0.05, **p < 0.01, ***p < 0.001.

To test the mediating role of social interaction in the effect of neighborhood environment on the health of residents, the mediating variable social interaction was added to Model 2 and Model 3 respectively, and the results were shown in Table 5. Model 4 shows the relationship between social interaction and the health of residents, and there is a significant positive relationship between social interaction and the health of residents, and for every 1-point increase in social interaction, the health of residents increases by 0.041 points. In Model 5, the mediator variable was added on the basis of Model 2. We find that both the social environment and social interaction have a significant positive effect on the health of residents. Compared with Model 2, the coefficient of the social environment’s effect on the health of residents decreases from 0.126 to 0.111 in Model 5, indicating that social interaction as a mediator variable can explain part of the effect of the social environment on the health of residents and plays a partial mediating role. Model 6 added the mediator variables based on Model 3. The results show that the physical environment and social interaction also have a significant positive effect on the health of residents. Compared to Model 3, the effect coefficient of the physical environment on the health of residents in Model 6 decreases from 0.104 to 0.103, which is a smaller decrease, but it can still be argued that social interaction plays a partial role as a mediator.

**Table 5:** Results of multiple regression analysis (mediating variable on residents’ health).

Variables	Model 4	Model 5	Model 6
Gender (male)	0.128** (0.045)	0.131** (0.045)	0.127** (0.045)
Marital status (married)	−0.002 (0.052)	−0.006 (0.052)	−0.011 (0.052)
Age	−0.023*** (0.002)	−0.023*** (0.002)	−0.023*** (0.002)
Education (higher)	0.227** (0.085)	0.248** (0.085)	0.195* (0.085)
Political profile (party member)	0.156* (0.073)	0.153* (0.073)	0.149* (0.073)
Occupation (institutional)	−0.070 (0.092)	−0.081 (0.092)	−0.074 (0.092)
Place of residence (urban)	0.163*** (0.050)	0.201*** (0.050)	0.117* (0.051)
The social environment		0.111*** (0.024)	
The physical environment			0.103*** (0.023)
Social interaction	0.041*** (0.010)	0.030** (0.010)	0.041*** (0.010)
F	48.79	46.48	46.28
P	0.000	0.000	0.000
R^2^	0.140	0.147	0.147

*p < 0.05, **p < 0.01, ***p < 0.001.

**Figure 2:**
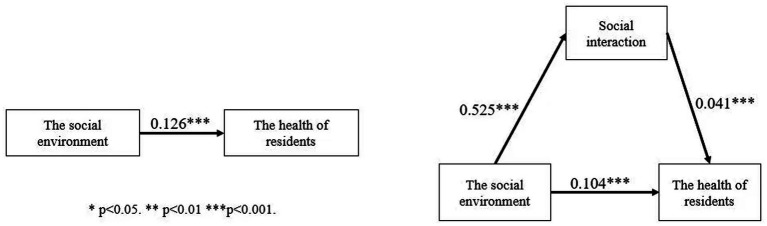
The mediation effect of social interaction.

To confirm whether social interaction plays a mediating role in the effect of neighborhood environment on the health of residents, the study further tested the relationship between neighborhood environment and social interaction, and the results were shown in Table 6. Model 7 shows that there is a significant positive effect of the social environment on social interaction, and for every 1-point increase in the social environment, the frequency of social interaction increases by 0.525 points. Combined with the results of the previous data analyses, Hypothesis 3 is confirmed, that is, the better the social environment, the higher the frequency of social interaction of the residents and, consequently, the better the health of residents (Figure 2). The positive influence between the physical environment and social interaction in Model 8 is not significant, indicating that social interaction does not play a mediating role in the relationship between the physical environment and the health of residents, and Hypothesis 4 is not verified. In addition, the insignificant effect of marital status on health in the model may be related to Chinese socio-cultural background. On the one hand, marriage may increase family responsibilities and economic stress, especially in rural areas, which may have a negative impact on residents’ health. On the other hand, unmarried groups may have certain advantages in economic independence and social support and further subgroups need to discuss the mechanism of health effects under different marital status.

**Table 6:** Multiple regressions of the mediating variable “social interaction”.

Variables	Model 7	Model 8
Gender (male)	−0.241** (0.085)	−0.267** (0.087)
Marital status (married)	−0.012 (0.098)	0.002 (0.101)
Age	−0.001 (0.003)	0.003 (0.003)
Education (higher)	−0.717*** (0.159)	−0.868*** (0.163)
Political profile (party member)	0.116 (0.137)	0.135 (0.141)
Occupation (institutional)	0.030 (0.173)	0.087 (0.177)
Place of residence (urban)	−0.176 (0.094)	−0.386*** (0.097)
The social environment	0.525*** (0.043)	
The physical environment		0.018 (0.044)
F	29.56	12.37
P	0.000	0.000
R^2^	0.090	0.040

*p < 0.05, **p < 0.01, ***p < 0.001.

### 4.3 Further analysis

In order to explore how the neighborhood environment affects the health of residents of different ages, the study divided the residents into three types according to age: the young, the middle-aged and the older adult. As shown in Table 7, the impact of neighborhood physical environment on health is the most obvious in the older adult group, followed by the middle-aged group, while for the young group, the impact of neighborhood physical environment on health is not significant. The neighborhood social environment has a significant impact on residents’ health of the three age groups and it is also the most obvious in the older adult group, followed by the youth group, and finally the middle-aged group. This may be due to the fact that the middle-aged people are busy with work, resulting in less time and frequency of their social interaction.

**Table 7:** Impact of neighborhood environment on the health of residents of different ages.

Age group	Neighborhood physical environment	Neighborhood social environment
The youth group (41 and below)	0.088	0.149***
The middle-aged group (42–71)	0.097**	0.093**
The older adult group (72 and above)	0.116*	0.156**

*p < 0.05, **p < 0.01, ***p < 0.001.

For the purpose of further exploring the role of social interaction in the impact of the physical environment on the health of residents, we refer to and draw on Preacher’s theory and method of testing mediation effect (128), this study operationalized social interaction into high, medium and low frequencies by ±SD to examine the impact of the physical environment on the health of residents at three different frequencies of social interaction. As shown in Table 8, the physical environment has a significant positive effect on the health of residents only at high and low frequencies of social interaction (*β* = 0.103, t = 2.43; β = 0.138, t = 3.90). For residents with low and high frequency of social interaction, the condition of the physical environment significantly promotes the health of residents; for residents with medium level of frequency of social interaction, the condition of the physical environment has no significant effect on the health of residents. The mediating effect of social interaction is significant between social environment and residents’ health, but not between physical environment and residents’ health. The possible reason is that the health effects of the physical environment are more direct, for example through the provision of exercise grounds and healthy food, while the mediating role of social interaction in the physical environment may be more important, which is also supported by the results of Model 7. We therefore conclude that social interaction plays a moderating role in the influence of the physical environment on the health of residents.

**Table 8:** Impact of physical environment on the health of residents under different frequencies of social interaction.

Level of moderating variables	Coefficient	Std. err.	t	*p*	95%CI	
Medium frequencies	0.065	0.043	1.51	0.133	−0.020	0.149
High frequencies (+1SD)	0.103	0.042	2.43	0.015*	0.020	0.187
Low frequencies (−1SD)	0.138	0.035	3.90	0.000***	0.069	0.208

*p < 0.05, **p < 0.01, ***p < 0.001.

## 5 Conclusions and discusses

### 5.1 Conclusion

The health of residents is the result of a combination of factors. This study places the health of residents in a community-level analytical framework and analyses the effects of the physical and social environment on the health of residents using data from the 2021 CGSS. The main conclusions are as follows: first, there is a strong relationship between neighborhood environment and the health of residents, and the better the neighborhood environment, the higher the level of the health of residents. When the neighborhood environment is further subdivided into the social environment and the physical environment, the results show that there is a significant positive correlation between both and the health of residents, which is in line with established research findings. Possible explanations are: (1) The quality of neighborhood environment itself represents the level of soc-economic status of the residents, and residents living in the better neighborhood environment are more likely to have higher soc-economic status, better material resources and greater health knowledge (129), which is more conducive to achieving higher levels of individual health. (2) The physical environment provides a place for residents’ daily life and leisure exercises, facilitating the proximity to purchasing goods, seeing doctors, exercising, and being close to nature, all of which have a positive impact on residents’ physical and mental health. (3) The favorable social environment is an important guarantee for residents to engage in social interactions, to accumulate social relationships and to receive social support, which helps them to maintain a positive and stable mood and to reduce the risk of illness, resulting in enhancement of the health of residents (130).

To further explore the influence mechanism of neighborhood environment on the health of residents, this study introduces the mediating variable of social interaction, and the regression results show that social interaction has a significant positive influence on the health of residents, and the social environment influences the health of residents by influencing social interaction, that is, the better the social environment is, the higher the frequency of residents’ social interaction will be, and the higher the level of the health of residents will be. Since there is a certain endogenous relationship between the social environment and social interaction, the data results suggest that the social environment and social interaction mediate each other in the relationship of influencing the health of residents, which has certain shortcomings in this study that need to be further explored in depth. While social interaction does not play a mediating effect in the influence of the physical environment on the health of residents. In order to further verify the influence of social interaction, this study divides social interaction into three different levels and examines the influence of the physical environment on the health of residents under different frequencies of social interaction, and finds that social interaction plays a moderating role in the effect of the physical environment on the health of residents. In particular, for residents with a low frequency of social interaction, the effect of the physical environment on their health is significant, which may be explained by the fact that a good physical environment promotes individual health behaviors in regard to residents with less social interaction, thus compensating for the decline in health caused by the lack of social interaction.

### 5.2 Recommendations

This study has confirmed the significant impact of neighborhood environment on residents’ health, which requires people to pay more attention to community construction and environmental governance (131), in order to create a more equitable and healthy community environment for residents and promote the accessibility of public services and further optimize the allocation of urban and rural resources, so as to further facilitate residents to carry out regular health activities and social interactions. Based on our research process and conclusions, we hereby put forward the following relevant suggestions: (1) Promote the equal allocation of community public facilities, especially in rural areas to strengthen the construction of community health facilities; (2) Encourage urban and rural community residents to participate in neighborhood interaction, for example, by organizing regular community activities and health lectures; (3) Optimize resources allocation and promote equal access to education and medical resources for urban and rural residents.

### 5.3 Limitations and future research prospects

The neighborhood environment is only one aspect of the factors affecting the health of residents. Whether and how to restrict the interrelated and interdependent factors is an important aspect of future research. However, the evidence presented in existing studies is often weak and the results have many inconsistencies, making it difficult to obtain definitive and conclusive opinions. In summary, the limitations of this study include: first, cross-sectional data cannot infer causality and future research can use longitudinal data for further verification. Second, social interaction variables are only measured by a single indicator, which may not be enough to fully capture the level of social interaction of residents. It is suggested that more subjective and objective indicators should be added to future research. Third, the model does not consider the interference of external variables such as culture and social norms, which may have a certain impact on the conclusion.

The relationship between neighborhood environment and residents’ health needs to be further studied in terms of data acquisition and model construction and needs to be further deepened and refined. Therefore, on the one hand, future studies should adopt more rigorous research design and strengthen the exploration of mediating pathways and effects. On the other hand, the specific indicators and analysis framework of the influence of neighborhood environment on residents’ health were established, and social experiments and structural interventions will be carried out in community units, so as to further explore the causal relationship between neighborhood environment and residents’ health. This is an important direction for our future research.

## Data Availability

The datasets presented in this study can be found in online repositories. Publicly available datasets were analyzed in this study. This data can be found here: http://cgss.ruc.edu.cn. Further inquiries can be directed to the corresponding author.
